# E+: Software for Hierarchical Modeling of Electron Scattering from Complex Structures

**DOI:** 10.1021/acs.jcim.5c00223

**Published:** 2025-05-07

**Authors:** Eytan Balken, Daniel Khaykelson, Itai Ben-Nun, Yael Levi-Kalisman, Lothar Houben, Boris Rybtchinski, Uri Raviv

**Affiliations:** † Institute of Chemistry, 26742 The Hebrew University of Jerusalem, Edmond J. Safra Campus, Givat Ram, 9190401 Jerusalem, Israel; ‡ Department of Molecular Chemistry and Materials Science, 34976 Weizmann Institute of Science, Rehovot 76100, Israel; § The Harvey M. Krueger Family Center for Nanoscience and Nanotechnology, The Hebrew University of Jerusalem, Edmond J. Safra Campus, Givat Ram, Jerusalem 9190401, Israel; ∥ Department of Chemical Research Support, Weizmann Institute of Science, Rehovot 76100, Israel

## Abstract

In modern nanobeam transmission electron microscopy methods, such as 4D-STEM, a converged electron nanobeam is scanned across a sample. Its 2D scattering pattern is recorded at each sample position, mapping the local sample structure. One of the bottlenecks in electron scattering is the analysis of the scattering data obtained from complex atomic or molecular structures. On the basis of D+ software, we developed the software E+ for analyzing electron scattering data, enabling us to model the 2D scattering pattern from any complex structure in a single orientation or a fiber. In addition, the azimuthally integrated 1D scattering curve of isotropically oriented structures (as in solutions or powders), or any other distribution of orientations, can also be computed. E+ allows the docking of geometric and/or molecular atomic models into their assembly symmetry. The assembly symmetry contains the rotations and translations of repeating subunits within a large structure. This process can be repeated hierarchically, using a bottom-up approach, adding as many subunits as needed. This procedure can be used to model the scattering data from any complex supramolecular structure at any spatial resolution, down to atomic resolution. In addition, the contribution from the solvation layers of structures in solutions can be computed in a scalable manner for large complexes. Furthermore, the Python API of E+ can be used for advanced modeling of structure factor and pair distribution functions, taking into account various effects, including thermal fluctuations, polydispersity of any structural parameters, or the intermolecular interactions between subunits. We validate E+ against the abTEM software and show a few examples, demonstrating how E+ can be used to analyze 4D-STEM electron scattering data.

## Graphical abstract

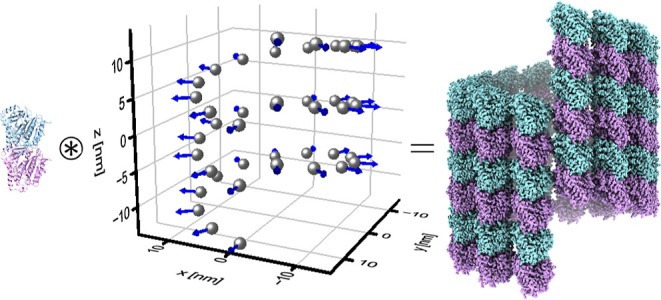

## Introduction

Transmission electron microscopy (TEM) is widely used for studying molecular structures and single particles in multiple operational modes. In four-dimensional scanning TEM (4D STEM), a converged electron nanobeam is scanned across a sample, and at each position, a 2D scattering pattern is recorded, mapping local crystal orientation, defects, crystallinity, and polymorphism.^1^ Multiple scattering can be reduced using nanobeam precision electron diffraction, where the diffraction from different tilt angles is averaged.^2^

One of the key bottlenecks in electron scattering is the analysis of data obtained from complex structures. Modeling approaches with adequate complexity, going beyond the independent atom models, are highly desirable in the field. The common atomistic modeling approaches are challenged when complex multicomponent molecular structures like ligand-capped nanoparticles, biological macromolecular structures, quasi-amorphous or partially ordered materials are under investigation.^3–6^

The recent reciprocal grid algorithm developed for X-ray scattering,^7–9^ allows the docking of atomic and/or geometric models^10–12^ into their assembly symmetry. The assembly symmetry includes the rotation angles and translation vectors of repeating subunits in a large structure. This process can be repeated hierarchically, applying a bottom-up approach, and adding as many different subunits as needed (Figure 1). This algorithm can compute the scattering intensity from any supramolecular structure at any spatial resolution (down to atomic models). In addition, the contribution of the excluded solvent and the solvation layer of structures in solutions can be computed in a scalable manner for large complexes. The effects of polydispersity in the dimensions of models, thermal fluctuation, intermolecular interactions, instrument resolution function, and radial distribution function (RDF) analyses can be quantitatively investigated (Figure 1).

X-ray and electron scattering experiments share similar fundamental principles. We therefore adapted the methodology of the D+ software, developed for X-ray scattering, for electron scattering, and created the E+ software. The advanced modeling algorithms developed for X-ray scattering were lacking in electron scattering and can immensely improve the quality of 4D-STEM data analysis, leading to previously unavailable structural insights. E+ contains all the features of D+, including its Python API, access to the GPU, and high parallelization, providing advanced modeling opportunities. Models can also be generated using rigorous computational methods and simulations. The models can be loaded into E+, which can compute their scattering patterns, and fitted to 4D-STEM scattering measurements. This process can be done iteratively until the models fit the data.

In the following sections, we explain how E+ computes the scattering amplitudes and intensities of complex structural models. We then validate E+ against abTEM software and analyze 4D-STEM measurements of graphene-monolayers, -bilayers, and -quadlayers, and *p*-mercaptobenzoic acid (*p*-MBA)-protected gold nanoparticles, comprising 144 gold atoms, Au_144_(*p*-MBA)_60_. In the last part, we analyze a large and complex microtubule model, containing many tubulin protein subunits.^13^

**Figure 1:**
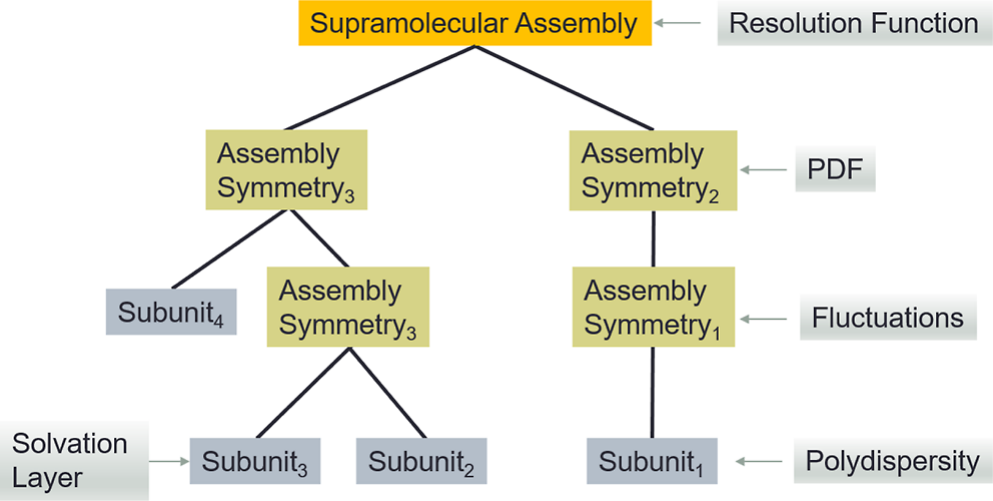
Hierarchical modeling approach of E+. A supramolecular structure can be modeled in a bottom-up approach from its assembling subunits. The subunits can be either atomic or geometric models, with or without a solvation layer, whose density slightly differs from the bulk solvent density. The polydispersity of each subunit dimension can be taken into account. Subunits can be docked into their assembly symmetries, describing how repeating subunits are shifted and rotated in space. This process can be repeated hierarchically; assemblies symmetries or subunits can be docked into other assembly symmetries, and the process can continue until the final structure is modeled. Effects such as thermal fluctuations in the structure or instrument resolution function can be taken into account at any relevant stage. In addition, based on the assembly symmetries, pair distribution analysis (PDF) can be performed. After the entire supramolecular structure is computed, the effect of the finite instrument resolution function can be taken into account.

## Materials and Methods

### Applied Theory

#### Electron Scattering

In electron scattering, the electron beam interacts with the atomic potentials, ϕ­(*r*), induced by the atoms present in the sample. Using the Schrödinger equation, the potential of these atoms can be numerically calculated.^14^ Once the potential is calculated, so can its scattering amplitude, *f* ^(e)^(*q*), through its Fourier transform in real space. As the potential is sphero-symmetric

1 $$f^{\left(e\right)}\left(q\right)=\frac{8\pi m_{0}e}{h^{2}}\int_{0}^{\infty }r^{2}\phi \left(r\right)\frac{\sin\left(qr\right)}{qr}dr$$

where *m*_0_ is the rest mass of the electron, *h* is Planck’s constant, $q=\frac{4\pi }{\lambda }\sin\theta$ is the magnitude of the scattering vector, *q⃗*, and θ is half of the scattering angle. Instead of *q*, used in E+, *k*

2 k≡θλ

or *s*

3 s≡q2π

are often used.^15,16^ A converter was built to switch between *q*, *s*, or *k* (Section Scattering Vector Converter).

The Mott–Bethe formula for neutral atoms^17,18^

4 $$f^{\left(e\right)}\left(q\right)=\frac{me^{2}}{2h^{2}}\frac{\left[Z-f^{\left(x\right)}\left(q\right)\right]}{q^{2}}$$

links the atomic form-factor obtained from electron scattering to the atomic form factor obtained from X-ray scattering

5 $$f^{\left(x\right)}\left(q\right)=4\pi r_{0}\int_{0}^{\infty }r^{2}\rho \left(r\right)\frac{\sin\left(qr\right)}{qr}dr$$

where m=m01−v2c2 for electrons of velocity *v*, *c* is the speed of light, *r*_0_ is the Thompson scattering length (2.82 pm), and ρ­(*r*) is the atom electron density at position *r*. In eq 4 and in D+, *r*_0_ is not included. We note that the Mott–Bethe equation for ions is slightly different, yet the conversion logic still holds. E+, however, goes beyond the Mott–Bethe approximation and computes the atomic form-factors as explained below.

#### Coordinate System

E+ uses the same Cartesian coordinate system used by D+, assuming the beam is aligned along the *y*-axis. This coordinate system should be kept in mind because it differs from the typical 4D-STEM experiment where the beam is parallel to the *z*-axis and the sample is put on top of a support grid parallel to the *xy*-plane. In E+ the equivalent plane would be the *xz*-plane.

#### Scattering Vector Converter

An accessory tool was built to convert from *q*, used in E+, to *k* (eq 2), using the following relations between them

6 $$q=\frac{4\pi }{\lambda }\sin\left(k\lambda \right)$$

and

7 k=arcsin(qλ4π)λ

The conversion between *q* and *s* uses eq 3.

#### Atomic and Atomic Group Form Factors

The atomic form factors can be more accurately approximated (compared with eq 4) using either the five-Gaussian^14,19^ or the five-Lorentzian approximation,^20^ or a combination of the two,^21^ used in the abTEM package.^5^ E+ (like D+)^8^ uses the five-Gaussian approximation

8 $$f^{\left(e\right)}\left(q\right)=\sum_{j=1}^{5}a_{j}e^{-b_{j}{\left(\frac{q}{40\pi }\right)}^{2}}$$

where the coefficients *a*_*j*_, *b*_*j*_ are those calculated by L.-M. Peng for neutral atoms and ions, which are accurate up to *q* ∼ 250 nm^–1^.^19,22^ In many biological proteins and lipids, the atomic groups SH, OH, CH_1/2/3_, and NH_1/2/3_ are often found and were therefore assigned specific symbols in the protein data bank file format (PDB). Hence, we fitted a five-Gaussian curve (eq 8) up to *q* = 250 nm^–1^ to the square root of the intensity received from Debye’s formula^23^ applied to those atomic groups

9 $$I\left(q\right)=\sum_{i=0}^{N-1}\left({\left|f_{i}^{\left(e\right)}\right|}^{2}+\sum_{j>i}^{N-1}2f_{i}^{\left(e\right)}f_{j}^{\left(e\right)}\frac{\sin\left(qr_{ij}\right)}{qr_{ij}}\right)$$

where *N* is the total number of atoms, *f*_*i*_^(e)^ and *f*_*j*_^(e)^ are the atomic form factors of the *i*-th and *j*-th atoms (eq 8), $r_{ij}\equiv \left|{\mathrm{r⃗}}_{i}-{\mathrm{r⃗}}_{j}\right|$ , and ${\mathrm{r⃗}}_{i}$ and ${\mathrm{r⃗}}_{j}$ are the positions of the *i*-th and *j*-th atoms in the atomic group. Table 1 shows the best-fitted (⟨RMSE⟩ = 0.025) five Gaussian coefficients of the atomic groups used in E+.

**Table 1:** Atomic Group Five Gaussian Coefficients That Best Fitted eq 9 up to *q* = 250 nm^–1^

atomic group	*a*_1_	*b*_1_	*a*_2_	*b*_2_	*a*_3_	*b*_3_	*a*_4_	*b*_4_	*a*_5_	*b*_5_
CH	0.1796	73.76	0.8554	5.399	1.75	27.15	0.05001	0.1116	0.2037	1.062
CH_2_	0.1575	89.04	0.8528	4.637	2.359	30.92	0.00496	–0.344	0.1935	0.6172
CH_3_	0.4245	4.092	0.4256	4.094	0.2008	74.32	2.884	33.65	0.16	0.4189
NH	0.1568	64.9	0.222	1.017	0.8391	4.656	1.469	23.17	0.05579	0.11
NH_2_	1.991	25.94	0.2351	74.54	0.8575	3.893	5.336	0.3422	–5.147	0.3388
NH_3_	–0.1646	168.7	0.2896	147.3	0.838	3.546	0.1736	0.4059	2.668	29.57
OH	0.1597	53.82	0.2445	0.7846	0.8406	4.042	1.235	20.92	0.03234	–0.01414
SH	–78.51	9.013	80.62	9.014	0.6401	1.924	2.665	37.71	0.2755	0.2941

By comparing the scattering amplitudes at *q* = 0 from X-rays with electrons, according to a material chemical formula and the corresponding atomic form factors, we created an accessory tool for converting the bulk electron density needed in D+ to the relevant zero potential, φ_0_, needed in E+.

#### Scattering of Molecules in Vacuum

The scattering amplitude from a molecule (in vacuum) is computed using its protein data bank (PDB) file representation, containing the positions ${\mathrm{r⃗}}_{j}$ and the type of each atom/atomic group in the molecule. The molecule should then be shifted so that its center of mass is at the origin to get more precise results for the same reciprocal grid size (determined by the number of shells in the spherical grid, defining the total number of precomputed scattering amplitude values and the spacing between them in the 3D reciprocal space representation).

Given a scattering vector in reciprocal space, *q⃗*, and a list of atoms and their coordinates (as in PDB files), the scattering amplitude of the entire molecular structure, containing *n* atoms, is given by

10 $$F_{mol}^{v}\left(\mathrm{q⃗}\right)=\sum_{j=1}^{n}f_{j}^{e}\left(q\right)\exp\left(i\mathrm{q⃗}\cdot {\mathrm{r⃗}}_{j}\right)$$

where ${\mathrm{r⃗}}_{j}$ is the location in real-space of the *j*th atom and *f*_*j*_^e^ is its atomic form factor, given by the five-Gaussian approximation (eq 8).

#### Scattering of Molecules in Solution

Molecules may be surrounded by a solvent and a solvation layer, whose local density and zero potential, φ_0_^Solvation Layer^, might be different from the zero potential of the bulk solvent, φ_0_. This is the case for 4D-STEM in liquid or frozen solvated samples at cryogenic temperatures (cryo 4D-STEM).

The scattering amplitude from PDB structures in solution can be computed in one of two ways. One option uses Dummy Atom Gaussian spheres to approximate the volume of solvent that is excluded by the atoms

11 $$F\left(\mathrm{q⃗}\right)=aF_{mol}^{v}\left(\mathrm{q⃗}\right)-\varphi_{0}f_{Excluded\;Solvent}^{Dummy\;Atom}\left(\mathrm{q⃗}\right)+\left(\varphi_{0}^{Solvation\;Layer}-\varphi_{0}\right)F_{Solvation\;Layer}\left(\mathrm{q⃗}\right)$$

Alternatively, the volume of excluded solvent can be taken into account as a collection of voxels

12 $$F\left(\mathrm{q⃗}\right)=aF_{mol}^{v}\left(\mathrm{q⃗}\right)-\varphi_{0}F_{Excluded\;Solvent}^{Voxel}\left(\mathrm{q⃗}\right)+\left(\varphi_{0}^{Solvation\;Layer}-\varphi_{0}\right)F_{Solvation\;Layer}\left(\mathrm{q⃗}\right)$$

*F*_mol_^*v*^ is defined in eq 10, *f*_Excluded_ _Solvent_^Dummy Atom^ in eq 17, *F*_Excluded_ _Solvent_^Voxel^ in eq 19, and *F*_Solvation Layer_ in eq 21. *a* is equal to 1 unless Solvent Only is indicated in E+, in which case *a* = 0.

Using the Python API of E+ and computer simulations, more advanced methods to compute the contribution of the solvent and the solvation layer can be applied (see, for example,^24^).

#### Solvent as Gaussian Dummy-Atoms

The mean atomic volume *V*_m_ = *N* ^–1^ *∑*_*j*_ *V*_*j*_ and mean atomic radius $r_{m}={\left(\frac{3}{4\pi }V_{m}\right)}^{1/3}$ are computed based on the list of atoms or atomic groups in the PDB file. *V*_*j*_ is the approximated volume of excluded solvent by the *j*th atom (or atomic group), computed based on the published experimental atomic radius, *r*_*j*_, of the *j*th atom (or atomic group),^25–28^ also used by D+.^8^

A Gaussian dummy atom is placed at the center of each atom in the PDB file, and its scattering amplitude is

13 $$F_{j}^{d}\left(q\right)=\varphi_{0}V_{j}\;\exp\left[-\frac{V_{m}^{2/3}q^{2}}{4\pi }\right]$$

where φ_0_ is the zero potential of the bulk solvent. *V*_m_ is used to uniformly adjust the volume of the excluded solvent throughout the entire structure, as also done in D+^8^ using

14 $$C_{1}\left(q\right)=c_{1}^{3}\;\exp\left[-\frac{V_{m}^{2/3}q^{2}\left(c_{1}^{2}-1\right)}{4\pi }\right]$$

where *c*_1_ is a fitting parameter whose default value is 1 and can vary slightly (up to 5%). The contribution of atom *j* to the scattering amplitude in solution is then

15 $$f_{j}^{s}\left(q\right)=f_{j}^{0}\left(q\right)-C_{1}\left(q\right)F_{j}^{d}\left(q\right)$$

The solution scattering amplitude from a molecule, given a list of *n* atoms, whose coordinates are ${\mathrm{r⃗}}_{j}$ , is

16 $$F_{mol}^{s}\left(\mathrm{q⃗}\right)=\sum_{j=1}^{n}f_{j}^{s}\left(q\right)\;\exp\left(i\mathrm{q⃗}\cdot {\mathrm{r⃗}}_{j}\right)$$

and the total excluded solvent contribution (the second term in eq 11) is

17 $$\varphi_{0}f_{Excluded\;Solvent}^{Dummy\;Atom}=C_{1}\left(q\right)\sum_{j=1}^{n}F_{j}^{d}\left(q\right)\;\exp\left(i\mathrm{q⃗}\cdot {\mathrm{r⃗}}_{j}\right)$$

E+ treats the contribution from hydrogen atoms as in D+,^8^ with the relevant table adjustments.

#### Voxelized Solvent

In this method, we equally divide the space occupied by the molecule into voxels of a predetermined size, *v* (whose default value is *v* = 0.2 nm). For each voxel, we determine whether it contains an atom (or part of one) or not by applying the algorithm of D+.^8^ The scattering amplitude of a voxel of dimensions, ω_*j*_, τ_*j*_, and μ_*j*_, in the *x*, *y*, and *z* directions, respectively, is^10^

18 $$f^{Voxel}\left(\mathrm{q⃗}\right)=\frac{8}{q_{x}q_{y}q_{z}}\sin\left(\frac{q_{x}\omega_{j}}{2}\right)\sin\left(\frac{q_{y}\tau_{j}}{2}\right)\sin\left(\frac{q_{z}\mu_{j}}{2}\right)$$

where the center of the *j*-th voxel is at ${\mathrm{r⃗}}_{j}^{Voxel}$ . The total scattering amplitude of the excluded voxels (the second term in eq 12) is the sum over the relevant voxels

19 $$F_{Excluded\;Solvent}^{Voxel}\left(\mathrm{q⃗}\right)=f^{Voxel}\left(\mathrm{q⃗}\right)\sum_{j\in \left\{\begin{array}{c} Exluded\\ Voxels \end{array}\right\}}\exp\left(i\mathrm{q⃗}\cdot {\mathrm{r⃗}}_{j}^{Voxel}\right)$$

and the scattering amplitude of the molecule in the solution (without the contribution of the solvation layer) is

20 $$F_{mol}^{sv}\left(\mathrm{q⃗}\right)=F_{mol}^{v}\left(\mathrm{q⃗}\right)-\varphi_{0}F_{Excluded\;Solvent}^{Voxel}\left(\mathrm{q⃗}\right)$$

#### Solvation Layer

To determine the scattering amplitude of the solvation layer, we sum over the scattering amplitudes from the collection of voxels comprising that solvation layer (the relevant voxels are identified as in D+^8^)­

21 $$F_{Solvation\;Layer}\left(\mathrm{q⃗}\right)=f^{Voxel}\left(\mathrm{q⃗}\right)\sum_{j\in \left\{\begin{array}{c} Solvation\\ layer\\ Voxels \end{array}\right\}}\exp\left(i\mathrm{q⃗}\cdot {\mathrm{r⃗}}_{j}^{Voxel}\right)$$

The scattering amplitude of the solvated molecule is

22 $$F_{Solvated\;Molecule}\left(\mathrm{q⃗}\right)=F_{mol}^{s}\left(\mathrm{q⃗}\right)+F_{Solvation\;Layer}\left(\mathrm{q⃗}\right)\left(\varphi_{0}^{Solvation\;Layer}-\varphi_{0}\right)$$

where $F_{mol}^{sv}\left(\mathrm{q⃗}\right)$ may be used instead of $F_{mol}^{s}\left(\mathrm{q⃗}\right)$ , if the excluded solvent volume was determined by the voxel method instead of dummy atoms.

The excluded solvent and solvation layer algorithms were thoroughly validated in our earlier solution X-ray scattering publications.^8,9,13,29,30^ These algorithms should be suitable for modeling cryo 4D-STEM data, after using the Electron Density Converter, to calculate the zero potentials of the solvent and the solvation layer (needed instead of the corresponding electron densities used in solution X-ray scattering).

#### Geometric Models

In the case where the exact molecular structure of a unit or a subunit is unknown, it is possible to calculate the scattering from a geometrical object similar to the subunit, as in D+,^8^ where instead of entering the electron density, like in X-ray scattering, one enters the zero potential, using our electron density converter module, which we have developed for this purpose (explained in Electron Density Converter). This potential is received, after proper normalization, from the value of the scattering amplitude at *q* = 0, just like the electron density is found in X-ray scattering amplitude at *q* = 0.

#### Hierarchical Models in E+

Models are defined in hierarchical data tree structures, with numbers of levels, nodes, or children, limited only by the computer’s capabilities. Geometric or atomic model subunits are the tree’s leaves. Repeating subunits, docked into their assembly symmetries, are the tree’s nodes, containing the locations and orientations of repeating subunits (Figure 1). The electron scattering amplitude of a supramolecular structure, containing *J* unique subunits, is

23 $$F\left(\mathrm{q⃗}\right)=\sum_{j=1}^{J}\sum_{m=1}^{M_{j}^{u}}\left[F_{j}\left(A_{j,m}^{-1}\mathrm{q⃗}\right)\sum_{k=1}^{K_{j,m}}\exp\left(i\mathrm{q⃗}\cdot {\mathrm{R⃗}}_{j,m,k}\right)\right]$$

where *M*_*j*_^u^ is the number of unique orientations of an object of type *j*, given by the Tait-Bryan rotation matrices **A**_*j*,*m*_. *K*_*j*,*m*_ is the number of real-space translations, ${\mathrm{R⃗}}_{j,m,k}$ , of object *j* with orientation **A**_*j*,*m*_.

E+ computes the scattering amplitudes of the subunits on 3D reciprocal-space grids. The reciprocal grids of larger structures at a higher level in the hierarchy are computed by interpolating the relevant neighboring (closest) precomputed lower-level surrounding reciprocal grid points. Repeating this process for all the leaves and nodes of the data tree structure leads to the final scattering amplitude.

In addition, the contribution of the solvation layer of structures in solutions can be computed in a scalable manner for large complexes, using the algorithms of D+.^8^

To compute the scattering from a complex structure, as done in D+, amplitudes can be summed hierarchically, using the direct, grid, or hybrid algorithms, as explained.^7^ This enables computing the scattering amplitudes and/or the intensity from very large structures, which is unavailable in other methods.^3–5^

#### Molecular Orientations

After the reciprocal grid electron scattering amplitude is computed, all the options for calculating the intensity of D+ are available in E+. This includes the solution orientation average, using all the integration methods of D+.

The 2D fiber diffraction intensity calculations (*get_fiber_intensity*, assuming a uniform distribution of azimuthal angles and a specific polar angle) and the 2D intensity from a single orientation (*get_crystal_intensity*, assuming a specific azimuthal angle and a specific polar angle) can be calculated using the Python API, as in D+.^9^ The user should specify the 2D intensity density by the total number of calculated points along each detector axis (providing the total number of points from the negative to the positive side of the detector). The same number of calculated points is used for both the *q*_⊥_ and *q*_*z*_ axes, where $q_{⊥}\equiv \sqrt{q_{x}^{2}+q_{y}^{2}}$ . Hence, the total size of the 2D intensity matrix will be the number of calculated points squared.

#### Other Angular Distributions

Using the Python API, other angular distributions may also be computed. After loading the scattering amplitude, azimuthal and polar angles in reciprocal space can be selected according to any distribution, Specifically, a Gaussian (*MC_gaussian_1D* and *MC_gaussian_2D*) and uniform distributions (*MC_uniform_1D* and *MC_uniform_2D*) of polar and/or azimuthal angles were implemented in the Python API.

#### Azimuthal Integration of 2D Scattering Patterns

To azimuthally integrate the 2D scattering patterns into a 1D scattering curve, the absolute *q*-value of each pixel was calculated

24 $$q=\sqrt{q_{⊥}^{2}+q_{z}^{2}}$$

The division resolution is Δ*q* = *q*_max_/*N*_*p*_, where *q*_max_ is the maximum detector *q*-range and *N*_p_ is the number of decided final *q*-points. The azimuthally integrated intensity is then

25 $$I\left(q_{i}\right)=\sum_{k}\frac{1}{N_{k}}I\left(q^{k}\right)$$

where *i* ∈ (1,···,*N*_p_) and *k* is the indexes of all the *N*_*k*_ detector pixels within the shell defined by

26 $$\left(i-1\right)\Delta q\leq q^{k}<i\Delta q$$

Specifically, we used Δ*q* = 0.2044 nm^–1^ and OriginLab’s dedicated binning function for this calculation.

#### Advanced Modeling Options

Using the Python API of E+ all the features of the Python API of D+, explained in our earlier papers,^8,9^ are available in E+. Particularly, the pair distribution function and the structure factor modules, including all the supporting functions, are available.^9^ Furthermore, the scattering amplitude of any structure can be computed outside the graphic user interface (GUI) of E+, loaded into E+, and serve as a subunit for computing the scattering amplitude of a larger structure. In particular, the scattering amplitude can be computed based on first principle simulations, including all-electron density functional theory (DFT), or any other method. This effort is worthwhile if high-resolution data (i.e., *q* > 50 nm^–1^) are available and can detect the contrast in the electronic structure of materials, including, for example, the redistribution of charge owing to chemical bonding.^4,31^

#### Instrument Resolution Function, and Polydispersity

A Gaussian instrument resolution function, characterized by a standard deviation, σ, can be applied in the GUI of E+ or the Python API, using the command apply_resolution. This function is suitable for diffraction-limited nanobeam setups having high momentum resolution.

Using the Python API of E+, the polydispersity of any geometric model parameter can be computed using a default Gaussian weighting function with 15 equally spaced sampling values, as done in X+^11^ or D+.^9^ Other weighting functions^32–34^ or the polydispersity of atomic models can be implemented through a more advanced usage of the Python API.

#### Ligand Swapping

To swap ligand A for ligand B, we first shifted ligand B to the origin. All the atoms *i* of molecule B were shifted by the position of the atom bound to the nanoparticle in B, ${\mathrm{r⃗}}_{Bound}^{B}$

27 $${\mathrm{r⃗}}_{i}^{0,B}={\mathrm{r⃗}}_{i}^{B}-{\mathrm{r⃗}}_{Bound}^{B}$$

where *i* ∈ B. We then define the vector ${\mathrm{r⃗}}_{start}^{0,B}$ , going from the nanoparticle-bound atom of molecule B to the most distant atom in the B molecule. The final direction of the B molecule is the direction of the A molecule, ${\mathrm{r⃗}}_{end}^{A}$ , similarly defined from the nanoparticle-bound atom of the A molecule to its most distant atom. We then rotated the shifted B molecule, ${\mathrm{r⃗}}_{i}^{0,B}$ from its initial direction, ${\mathrm{r⃗}}_{start}^{0,B}$ , into the direction of the A molecule, ${\mathrm{r⃗}}_{end}^{A}$ around the rotation axis

28 $${\mathrm{r⃗}}_{rot\;axis}=\frac{{\mathrm{r⃗}}_{start}^{0,B}\times {\mathrm{r⃗}}_{end}^{A}}{\left|{\mathrm{r⃗}}_{start}^{0,B}\times {\mathrm{r⃗}}_{end}^{A}\right|}$$

by the rotation angle

29 $$\theta =\arccos\left(\frac{{\mathrm{r⃗}}_{start}^{0,B}\cdot {\mathrm{r⃗}}_{end}^{A}}{\left|{\mathrm{r⃗}}_{start}^{0,B}\right|\left|{\mathrm{r⃗}}_{end}^{A}\right|}\right)$$

Using Rodrigues’ rotation formula^35^ we computed the rotated molecule B

30 $${\mathrm{r⃗}}_{i}^{rot,B}={\mathrm{r⃗}}_{i}^{0,B}\cos\theta +\left({\mathrm{r⃗}}_{rot\;axis}\times {\mathrm{r⃗}}_{i}^{0,B}\right)\sin\theta +{\mathrm{r⃗}}_{rot\;axis}\left({\mathrm{r⃗}}_{rot\;axis}\cdot {\mathrm{r⃗}}_{i}^{0,B}\right)\left(1-\cos\theta \right)$$

Finally, we translated the rotated B molecule so that its nanoparticle-bound atom is at the position of the nanoparticle-bound atom of molecule A

31 $${\mathrm{r⃗}}_{i}^{final,B}={\mathrm{r⃗}}_{i}^{rot,B}+{\mathrm{r⃗}}_{Bound}^{A}$$

where *i* ∈ B.

### Modules in E+

In addition to all software modules of D+, explained in our earlier papers,^8,9^ E+ has an Electron Density Converter, explained below. We also briefly mention Suggest Parameters and PDBUnits, which are very useful tools when using E+ or D+.

#### Electron Density Converter

As explained, some of the models or abilities in D+ depend on the material’s electron density. In electron scattering, instead of using the electron density, one should use the atomic zero potential, given by the electron scattering at *q* = 0. Thus, we have built a module whose sole function is to receive a bulk electron density, ρ_bulk_, a PDB file or a list of *N* atom and ion types, *i*, and their occurrences, *n*_*i*_, according to the molecule’s chemical formula, and return the corresponding zero potential. This conversion uses the five-Gaussian approximation coefficients of electrons ( ${\left(a_{i}\right)}_{j}^{e}$ , eq 8) and X-rays ( ${\left(a_{i}\right)}_{j}^{x}$ ,^8^) for atom type *i*. We then find the proportion coefficient using

32 $$p=\frac{\sum_{i=0}^{N-1}\left(n_{i}\sum_{j=1}^{5}{\left(a_{i}\right)}_{j}^{x}\right)}{\sum_{i=0}^{N-1}\left(n_{i}\sum_{j=1}^{5}{\left(a_{i}\right)}_{j}^{e}\right)}$$

and the zero potential is then easily found

33 $$\varphi_{0}=\frac{\rho_{bulk}}{p}$$

The returned value (in units of e^−^/nm) can now be used to calculate the scattering amplitude of either geometric models, the solvent-excluded volume, or the contribution of the solvation layer surrounding a molecule.

#### Suggest Parameters and PDBUnits

Suggest Parameters and PDBUnits are accessory tools that were created for D+ and are available in E+. These tools were explained in our earlier paper.^8^ Briefly, Suggest Parameters gets the dimensions of the computed model and provides the relevant computational parameters, such as the size of the grid and integration parameters, needed for correctly computing the model. PDBUnits gets a PDB file of a subunit and a PDB file containing several repeating subunits and finds all the positions and orientations (a docking list file or a *dol* file) of all the repeating subunits in the latter PDB file.

### Experimental Section

#### 4D-STEM Measurements

4D STEM data sets were obtained in a double aberration-corrected Themis-Z microscope (Thermo Fisher Scientific Electron Microscopy Solutions, Hillsboro, USA) equipped with a high-brightness field emission gun at an acceleration voltage of 200 keV. For the diffraction recording, an electron probe with a convergence angle of 0.2 mrad was adjusted in STEM microprobe mode with a real space probe size of about 6 nm in diameter. A primary beam current between 1 and 4 pA was used. An electron microscope pixel array detector (Cornell/FEI EMPAD) with 128 × 128 pixels^36,37^ allowed rapid data collection of the entire unsaturated diffraction pattern with a single frame of 1 ms for each pattern.

#### X-ray Scattering

Solution small- and wide-angle X-ray scattering (SAXS and WAXS) measurements of gold nanoparticles shown in Figures 4 and S1 were done in our in-house X-ray scattering setup, described elsewhere.^38^ SAXS measurements in Figure 5 were performed at the ID02 beamline at the ESRF.^39,40^

**Figure 2:**
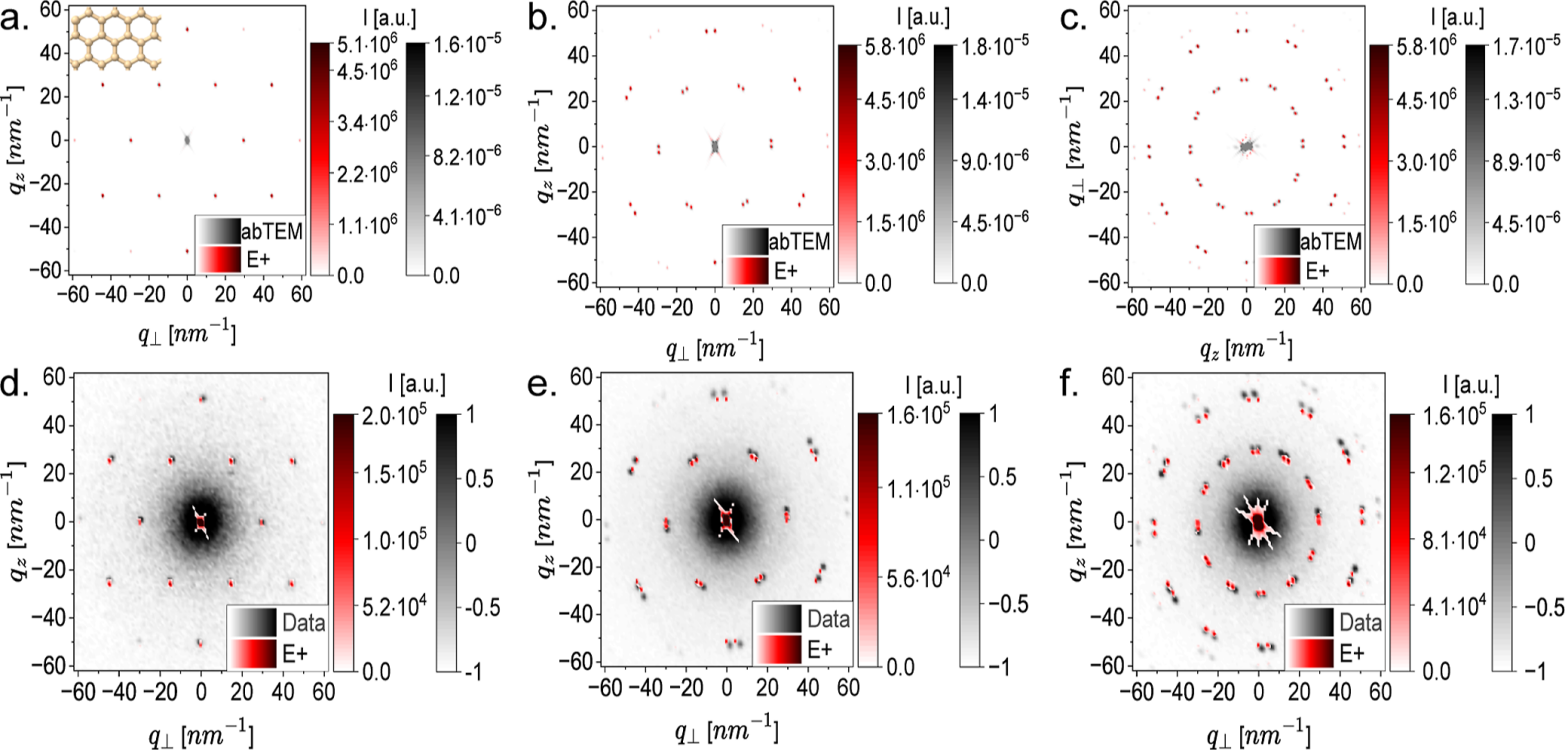
Graphene monolayer, bilayer, and quadlayer. (a). An atomic model of a hexagonal graphene monolayer lattice with 25 × 25 repeating subunits, perpendicular to the *y*-axis (the beam axis) as computed by E+ (red), overlapping a similar graphene monolayer, perpendicular to the *z*-axis (the beam axis) as computed by abTEM (gray). (b) The E+ computed 2D scattering pattern of a graphene bilayer (red) overlapped with the calculation of a similar bilayer calculated by abTEM (gray). The bilayers were parallel to the *xz*-plane for the E+ calculations and to the *xy*-plane for the abTEM calculations. The spacing between the graphene monolayers, taken from panel (a), was 0.348 nm and the top monolayer was rotated by β = 5° around the beam axis (*y* or *z*-axis for E+ or abTEM, respectively). (c) The E+ computed 2D scattering pattern of graphene quadlayer lattices at perpendicular orientation with respect to the *y*-axis (red) overlapped with the calculation of a similar graphene quadlayer parallel to the *xy*-plane, calculated by abTEM (gray). In E+, the hierarchical modeling was based on the bilayer from (b) with a rotation of β = 30° and a spacing of 0.348 nm between the two bilayers, aligned parallel to the *xz*-plane. (d) An average of 22 4D-STEM measurements of graphene monolayers (gray) compared with uniformly averaged E+ atomic models of graphene monolayers with hexagonal lattices containing between 15 × 15 and 26 × 26 subunits in positional correlation (red). (e) An averaged 4D-STEM measurement from a graphene bilayer at perpendicular orientation with respect to the beam axis (gray) compared with a uniformly averaged E+ 2D scattering pattern of graphene bilayers (as in panel b), where each monolayer contains between 15 × 15 and 26 × 26 graphene subunits (red). (f) An averaged 2D electron scattering pattern from a graphene quad-layer at perpendicular orientation with respect to the beam axis (gray) versus the computed E+ averaged model of graphene quadlayers with a rotation of β = 30° between the two bilayers (similar to panel c) parallel to the *xz*-plane, where each monolayer contains between 15 × 15 and 26 × 26 graphene subunits (red).

**Figure 3:**
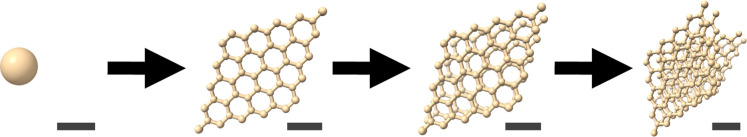
Hierarchical modeling of the graphene models, from a single carbon atom (leftmost) to the quadlayer (rightmost). The scale bar equals 0.51 Å for the leftmost figure and 2 Å for all the other models. Molecular pictures were made using UCSF ChimeraX^45^

**Figure 4:**
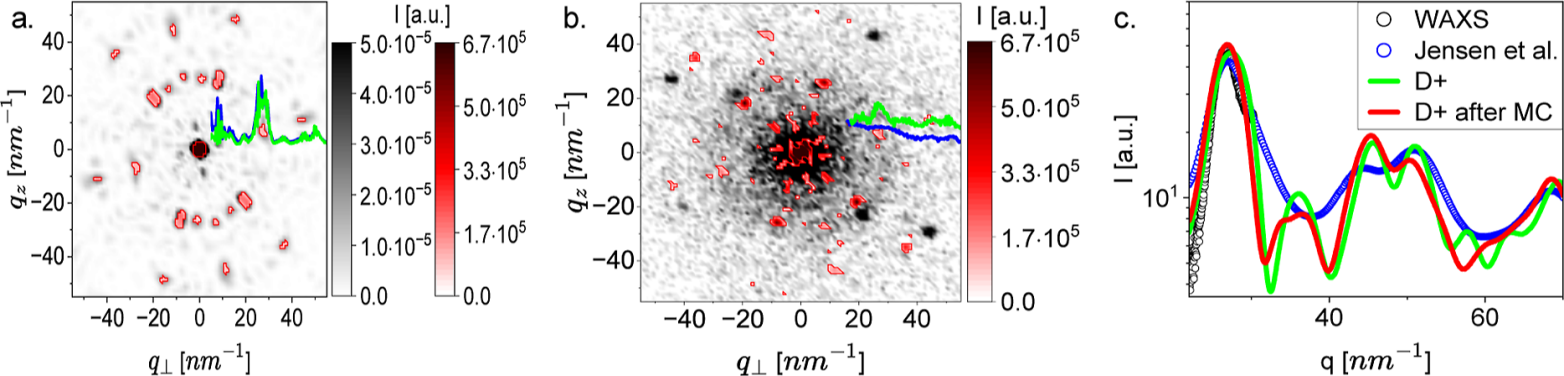
Gold Nanoparticles. (a) The computed 2D scattering pattern from the initial configuration of the Au_144_(*p*-MBA)_60_ nanoparticle (see text) computed by abTEM (grayscale), overlapped with the computed 2D scattering pattern computed by E+ (red). The 2D patterns were azimuthally averaged as explained in Azimuthal Integration of 2D Scattering Patterns, using OriginLab’s binning function, with Δ*q* = 0.7 nm^–1^ (blue, abTEM) and Δ*q* = 0.4 nm^–1^ (green, E+). (b) A 4D-STEM scattering pattern from a single Au_144_(*p*-MBA)_60_ nanoparticle on a graphene grid under vacuum (grayscale) and the computed averaged E+ model (red-scale) following MC simulations, as explained in the text. The measured and computed 2D patterns were azimuthally averaged as explained in Azimuthal Integration of 2D Scattering Patterns, using OriginLab’s binning function, with Δ*q* = 0.4 nm^–1^ for both the measurement (blue) and E+ (green). (c) Background-subtracted azimuthally integrated wide-angle X-ray scattering (WAXS) from Au_144_(*p*-MBA)_60_ nanoparticles in solution (black open symbols), previously published X-ray powder diffraction measurement^48^ (blue open symbols), the calculated X-ray scattering curve (using D+) from the averaged Monte Carlo simulated nanoparticle atomic models (red curve), and the calculated X-ray scattering curve of the initial gold-NP model (green curve).

**Figure 5:**
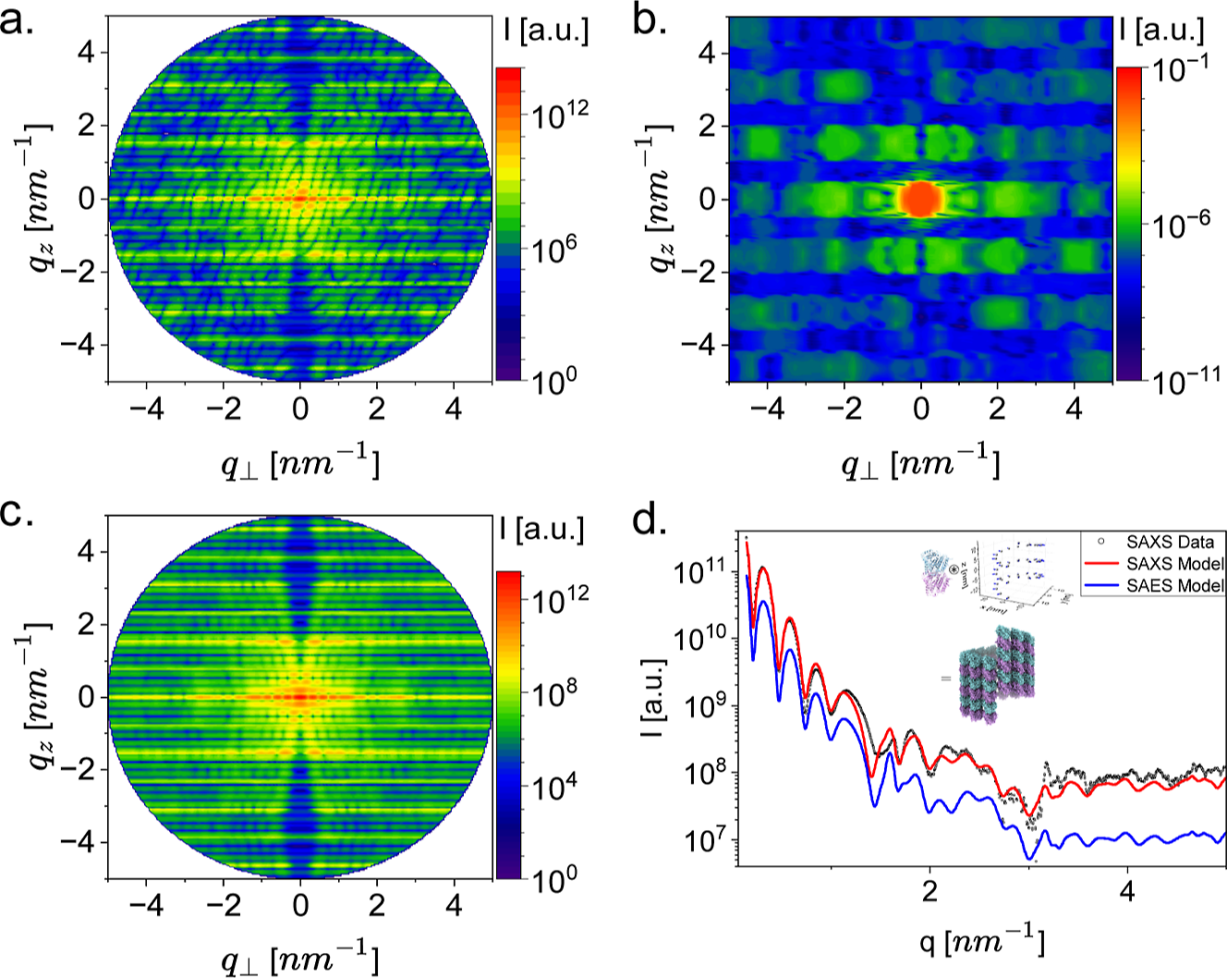
Scattering from microtubules. The microtubule model was created by docking the atomic model of a tubulin dimer (PDB ID 3J6F) onto a 3-start left-handed helical lattice with a pitch of 12.214 nm, and a radius of 11.9 nm to the geometric center of the dimer atomic coordinates. This model corresponds to a microtubule with 14 protofilaments. Each protofilament contained 16 tubulin dimers. The scattering intensities are shown on logarithmic scales next to each 2D pattern. (a) The 2D electron scattering pattern computed by E+, from the atomic model of an oriented 14 protofilament microtubule whose long axis is parallel to the *z*-axis. (b) The 2D electron scattering from the same oriented microtubule computed by the abTEM program.^5^ (c) The expected 2D electron fiber diffraction from the same 14-protofilament microtubule, computed by E+. (d) Background-subtracted solution small-angle X-ray scattering (SAXS) data of microtubule measured as explained in Microtubule (symbols). The data were adapted from^7^ and fitted to a weighted-averaged microtubule model, with radii of 11.05, 11.9, and 12.75 nm to the geometric center of the dimer atomic coordinates, corresponding to 13, 14, and 15 protofilaments. The mass fraction of tubulin in the models is 0.2, 0.7, and 0.1, respectively. The SAXS model was computed by D+ (red curve).^13^ The solution small-angle electron scattering (SAES) of the same structure was computed by E+ (blue curve). The models took the solution (water) surrounding the protein into account using the voxel method (called Dummy Atoms (voxelized)). In D+, the electron density of water, 333*e* ^–^/nm^3^, was used. In E+, we used its zero-potential, 101.27*e* ^–^/nm, as calculated by the electron density converter Electron Density Converter. The solvent voxels had a size of 0.05 nm. The inset graphically shows how the convolution of the αβ-tubulin dimer with the left-handed helical lattice creates the microtubule structure.

### Materials

#### Graphene

A single layer PELCO Graphene TEM Support Films, suspended on a lacey carbon film, 300-mesh copper grid, was purchased from TED PELLA Inc., and used after passivation. In a few cases, the films contained 2, 3, or even 4 graphene sheets.

#### Gold Nanoparticles

We synthesized *p*-mercaptobenzoic acid (*p*-MBA)-protected gold nanoparticle, comprising 144 gold atoms, Au_144_(*p*-MBA)_60_ as previously described.^41^

#### Microtubule

Tubulin was purified as explained.^42^ The microtubule sample was prepared and measured as explained.^7^ Briefly, 20 mg/mL tubulin in BRB80 buffer supplemented with 4 mM guanosine-5′-triphosphate (GTP) was incubated at 25 °C for 30 min. The resulting microtubule solution was measured at the ID02 SAXS beamline of the ESRF. The sample was then centrifuged at 20800 g at 25 °C for 30 min, and the supernatant, containing coexisting small tubulin assemblies and dimeric tubulin, was measured at the same spot in the flow-cell capillary. The scattering curve of the supernatant served as a background for the microtubule measurement, as explained.^13^

## Results and Discussion

### Graphene Layers

As graphene grids are often used to perform 4D-STEM measurements, we first analyzed graphene monolayers, bilayers, and quadlayers, observed in earlier^43,44^ and our experiments (Figure 2). Using E+, we computed a hexagonal graphene monolayer lattice with 25 × 25 repeating subunits in positional correlation, aligned along the *xz* plane, perpendicular to the electron beam directed along the *y*-axis (see Coordinate System). E+ builds molecular models by placing each atom at its position in the molecular structure (eq 10). The model computed by E+ adequately agreed with a similar model, computed by abTEM^5^ (Figure 2a). The model calculated by abTEM used the same model as E+ (exported as a PDB) after a 90° rotation around the *x*-axis to be perpendicular to the *z*-axis, which is the beam axis in abTEM.

Bilayers of graphene were previously observed and characterized by X-ray measurements, which revealed a spacing of 0.348 nm between the two graphene monolayers.^44^ We used our 25 × 25 graphene monolayer model and added a second vertically shifted monolayer around the beam axis, using the “Manual Symmetry” option of E+ (Figure 3). We then slightly varied the rotation (or twist) of the second graphene monolayer around the electron beam axis by 5° (Figure 2b). We then validated the 2D scattering pattern computed by E+ with that of abTEM. The same protocol was repeated to generate a quadlayer model, but we added a 30° rotation and a gap of 0.348 nm between the two bilayers­(Figure 2c).

To compare with our 4D-STEM measurements of graphene monolayers, we varied in E+ the number of hexagonal subunits in positional correlation in the graphene lattices between 15 × 15 and 26 × 26. We then uniformly averaged the scattering intensities of the series of lattices (assuming each had an equal weight) and compared the computed averaged E+ intensity with the averaged intensity of 22 4D-STEM graphene monolayer measurements, performed at different positions across a graphene TEM support film (Figure 2d). In some of our experiments, we also observed graphene bilayers and quadlayers (Figure 2e and f, grayscale) and compared them with similarly averaged models computed by E+ (Figure 2e and f, red).

Whereas the fit between E+ and the 4D-STEM data is adequate, there are still small differences between our models and the data. These differences could be modeled by considering more rigorous physical models of graphene bi/quad-layers, as was recently done when analyzing 4D-STEM data from graphene bilayers, initially prepared with a small twist between their monolayers.^43^ This claim is supported by the good agreement between the scattering patterns computed by E+ and abTEM (Figure 2a–c).^5^

### Gold Nanoparticles

To demonstrate more advanced capabilities of E+, we investigated *p*-mercaptobenzoic acid (*p*-MBA)-protected gold nanoparticle, comprising 144 gold atoms, Au_144_(*p*-MBA)_60_ (Figure 4). This gold nanoparticle belongs to an important class of materials with properties between molecules and particles.^46,47^ It was characterized by the atomic pair distribution function (PDF) of X-ray powder diffraction data,^48^ but its structure determination by X-ray crystallography has not been achieved yet. Nevertheless, the structure of a similar particle, Au_144_(SCH_2_Ph)_60_, was solved by X-ray crystallography, CSD Entry: TIRBAA.^49^

To model 4D-STEM measurements from Au_144_(*p*-MBA)_60_, we used the published structure of Au_144_(SCH_2_Ph)_60_,^49^ and replaced the SCH_2_Ph ligands with *p*-MBA ligands. The structure of the *p*-MBA ligand was based on the structure of a similar particle, Au_102_(*p*-MBA)_44_, elucidated by X-ray crystallography^50^ and further characterized by transmission electron microscopy at cryogenic temperatures (Cryo-TEM),^51^ NMR,^52^ and solution X-ray scattering.^53^

The ligand exchange was done by finding the starting orientation vector of the *p*-MBA molecule (vector from the S-atom, bound to a gold atom on the surface of the nanoparticle, toward the farthest H-atom) and its ending orientation vectors (that of the SCH_2_Ph molecule, similarly determined). The *p*-MBA vector was then rotated to all the ending vectors using Rodrigues’ rotation formula^35^ as explained in Ligand Swapping. Our model assumed that the 144 gold atoms and the *p*-MBA ligands kept the arrangement of the SCH_2_Ph ligands. This assumption, however, is an approximation.^48^

Using E+, we computed the 2D scattering pattern from the above model after a rotation of 35° about the *y*-axis (Figure 4a). The rotation was applied to match the experimental particle’s orientation with respect to the electron beam (Figure 4b). Similarly, to match the experimental electron scattering *q*-range, the models were computed up to *q*_max_ = 70 nm^–1^. In addition, to match the resolution of our detector, a resolution of 128 × 128 pixels was computed for the 2D diffraction pattern (Figure 4a and b). The result of E+ agreed with abTEM (Figure 4a). The agreement was further validated by azimuthal integration of the 2D patterns into 1D curves shown on top of the patterns (Figure 4a).

To compare with 4D-STEM and X-ray scattering experimental data (Figure 4b,c), we started from the above model (Figure 4a) and applied more advanced options of E+. Using the Python API of E+, we ran Monte Carlo simulations (*MC_Sim*) that took into account the interactions between gold atoms and the effect of thermal fluctuations. In the simulations, the gold atoms of the nanoparticle interacted through a Lennard-Jones potential

34 $$V_{LJ}\left(r\right)=4\epsilon \left[{\left(\frac{\sigma_{ev}}{r}\right)}^{12}-{\left(\frac{\sigma_{ev}}{r}\right)}^{6}\right]$$

at a finite temperature (298 K). We examined the effect of varying the depth of the attractive well ϵ at a fixed excluded volume σ_ev_ of 0.27 nm (Figure S1a) and the effect of varying σ_ev_ at a fixed ϵ of 0.4106ev = 16.079*k*_B_ *T* (Figure S1b). After the simulations attained steady-state, we selected 200 atomic gold nanoparticle accepted configurations. For each configuration, we computed its scattering amplitude and added it to the scattering amplitude of the ligands at their original configuration. It is interesting to note that electron scattering is more sensitive to the contribution of the ligands than X-ray scattering (Figure S2). We then computed the 2D intensity pattern and the 1D intensity curve in a solution for each accepted configuration in the Monte Carlo simulation. The 1D curve was obtained after computing the orientation average in reciprocal space, assuming an isotropic distribution of particles in all orientations, as in a solution. Finally, we averaged the 2D (Figure 4b) and 1D scattering intensities from all the simulated nanoparticle configurations and compared them with 4D-STEM experimental (Figure 4b) and solution X-ray scattering data (Figure 4c). To match the experimental 1D curves, a resolution of 3500 points was computed (Figures 4c and S3). The 1D data were compared with our solution wide-angle X-ray scattering (WAXS) data and earlier X-ray powder diffraction data.^48^ By comparing with the 4D-STEM (Figure 4b) and X-ray scattering data (Figure 4c) we found a best-fitted excluded volume term of σ_ev_ = 0.268 nm (corresponding to a mean steady-state bond length of 0.301 nm) and a best-fitted attractive well depth of ϵ = 0.4129 eV = 16.079*k*_B_ *T*.^54^

The overlap between the measurements and the computed average 2D electron scattering of accepted Monte Carlo configurations is adequate at the first set of clearly resolved peaks, corresponding to the spacing between the centers of nearest neighbor gold atoms (Figure 4b). At higher *q* values, the computed model deviates from the data. Deviations from the model were also observed at the lower *q*-values measured with our in-house X-ray scattering setup,^38^ showing a shift of the first minimum in the scattering curve to a lower *q*-value, suggesting an increase in the nanoparticle mean radius (Figure S3).

The computed solution X-ray scattering curve of the gold nanoparticle initial configuration is rather close to the peak corresponding to the spacing between the centers of nearest neighbor gold atoms (Figure 4c, green curve at *q* ≈ 27 nm^–1^). After averaging the azimuthally integrated scattering intensity of all the accepted Monte Carlo configurations, we observe adequate overlap at the nearest neighbor gold atom peak of the computed model, the scattering curve from nanoparticles measured in our in-house X-ray scattering setup, and the published X-ray powder diffraction data (Figure 4c, red, black, and blue curves, respectively).^48^

To determine the shape of our gold nanoparticles in solution, we used the X+ program^10,11^ to analyze the azimuthally integrated background-subtracted solution X-ray scattering data (Figure S3). We fitted the data to a core–shell spherical model with a gold core radius of 1.12 nm, polydispersity with a variance, σ^2^, of 0.096 nm, and a mean core electron density of 5560*e* ^–^/nm^3^ ^53^ and a ligand shell with a thickness of 1.048 nm and a mean shell electron density of 260*e* ^–^/nm^3^. X+ calculates polydispersity using a Gaussian distribution of 15 radii around the mean radius according to the σ value. The fit between the data and the model is adequate (Figure S3), showing that small-angle scattering data provides additional structural insight to earlier models.^48^ It also shows that the nanoparticles had some polydispersity in their size, meaning the nanoparticles most likely formed larger particles (i.e., with more than 144 gold atoms per particle). Assembly of the Au_144_(*p*-MBA)_60_ nanoparticles into dimers or trimers did not explain our low-angle data (Figure S3).

We realize that our gold nanoparticle model is inaccurate, most likely owing to our crude assumptions. However, the analysis of the Au_144_(*p*-MBA)_60_ nanoparticles demonstrated how E+ can be used to compute and test sophisticated models. Resolving the exact structure of this specific gold nanoparticle sample is beyond the scope of this paper, focusing on the E+ program.

### Complex Hierarchical Structures

One of the important advantages of E+ is the modeling of large, complex hierarchical structures. To demonstrate this power, we computed the electron scattering from microtubules (Figure 5). Microtubule filaments are found in all eukaryotic cells and play an important role in cell division, organelle transport, and cell motility. Microtubule is a protein polymer made of many copies of αβ-tubulin heterodimers, assemble head-to-tail into straight protofilaments, which then assemble laterally into hollow nanotubules, typically containing between 13 and 15 protofilaments (depending on the assembly conditions).^13,58^ The microtubule structure can also be created by docking tubulin dimers onto a discontinuous (owing to the seam) left-handed helical lattice (Figure 5d, inset).^55^ Cryo-TEM resolved the atomic microtubule structure,^56,57^ and the structure is consistent with solution X-ray scattering data.^7,8,13^

We compared the 2D scattering pattern from a single orientation of an atomic microtubule model built out of 14 protofilaments, each containing 16 dimers aligned along the *z*-axis, computed by E+ (Figure 5a) and abTEM (Figure 5b). The 2D scattering pattern of E+ shows the expected oscillations in the equatorial direction, corresponding to the tubule radius. In the meridional direction, we get the layer lines corresponding to the pitch of the helical arrangement of the subunits. In addition, the cross pattern is forming because the maximum intensity of the higher-ordered Bessel functions (the Fourier Transform of the *n*-th helical turn) is shifted to higher *q*_⊥_ values. The slope of the cross shape is also a function of the tubule radius (Figure 5a). The 2D scattering pattern of abTEM (Figure 5b), gives a crude representation of the expected pattern.

Using the Python API of E+ (*get_fiber_intensity*), we computed the expected electron scattering from a fiber of microtubules (Figure 5c). The features observed in the single orientation became sharper and clearer in the fiber diffraction owing to the azimuthal angle average in reciprocal space.

Finally, we computed the expected electron scattering curve from a microtubule solution (Figure 5d, blue curve). The latter was compared with an X-ray scattering measurement from a microtubule solution (Figure 5d, symbols) and the corresponding X-ray model, computed by D+ (Figure 5d, red curve).^8,9^ In solution, the models computed by D+ and E+ contained a linear combination of microtubules with 13, 14, and 15 protofilaments, built out of 16 tubulin dimers each, where the mass fraction of tubulin in the models was 0.2, 0.7, and 0.1, respectively (Figure 5d). The models also took into account the solvation layer surrounding the protein using the voxel method. In D+, the electron density of water, 333*e* ^–^/nm^3^, was used. In E+ we used its zero-potential, 101.27*e* ^–^/nm, as calculated by the electron density converter. The voxels had a size of 0.05 nm. While the electron scattering of the microtubule was not measured, the small-angle electron scattering (SAES) model was compared to match its solution X-ray scattering counterpart model and measurement. The E+ model has all the X-ray features, including minima and maxima locations and a sharper decay at higher *q*-values (Figure 5d), as expected (eq 4). We note that this model could have been quickly calculated thanks to its clear hierarchy, where a single αβ-heterotubulin dimer was docked into the ordered helical structure, making it perfect for the E+ reciprocal grid algorithm and the Hybrid method.^7,8^

## Conclusions

E+ is a versatile software for analyzing electron scattering data from any complex structure in a single orientation, fiber, or random orientation, in a vacuum or solution. E+ was validated against the abTEM software and experimental data. It may be integrated into current electron microscopy methodology, particularly 4D STEMs, and Cryo-4D STEMs, immensely improving data analysis opportunities, and leading to powerful structural insights. As demonstrated here, E+ can be integrated with Monte Carlo simulations and account for the effects of intermolecular interactions on the observed scattering data. Similarly, other computational approaches like molecular dynamics simulations or density functional theory (DFT) calculations may be integrated with E+, using its Python API. Multiple scattering effects, however, are not taken into account in E+, hence, the analysis of thick (>100 nm) samples might be more challenging. Multiple scattering can be reduced using nanobeam precision electron diffraction, where the diffraction from different tilt angles is averaged, or electrons with higher energies.^2^

## Supplementary Material



## Data Availability

For academic usage, the code, E+ software, and its user’s manual (including an extensive Python API section) are available for download on the laboratory’s GitHub Page (https://github.com/uri-raviv-lab/dplus-dev/releases/tag/dplus-v5.1.6.0). The data and code used to create the figures of this paper are available on a separate GitHub repository (https://github.com/uri-raviv-lab/E_plus_paper).

## Notes

Three additional figures are available in the Supporting Information. Effects of the Lennard-Jones potential parameter values. The ligand contribution to the X-ray and electron scattering curves. Fit of the gold-nanoparticle scattering curve to a spherical model at low scattering angles and to its PDB model at wider scattering angles (PDF)
