# Supplementary material for: E+: Software for Hierarchical Modeling of Electron Scattering from Complex Structures
Source: J Chem Inf Model. 2025 May 7;65(10):4968–79. doi: 10.1021/acs.jcim.5c00223 (PMC12117565; doi:10.1021/acs.jcim.5c00223)
Supplement: Supplementary file 1 [file ci5c00223_si_001.pdf]

Supporting Information: E+: Software for hierarchical modeling of electron scattering from complex structures

Eytan Balken,[†,‖] Daniel Khaykelson,[‡,‖] Itai Ben-Nun,[†] Yael Levi-Kalisman,[¶] Lothar Houben,[§] Boris Rybtchinski,[‡] and Uri Raviv[*,†,¶]

†*Institute of Chemistry, The Hebrew University of Jerusalem, Edmond J. Safra Campus, Givat Ram, 9190401, Jerusalem, Israel*

‡*Department of Molecular Chemistry and Materials Science, Weizmann Institute of Science, Rehovot 7610001, Israel*

¶*The Harvey M. Krueger Family Center for Nanoscience and Nanotechnology, The Hebrew University of Jerusalem, Edmond J. Safra Campus, Givat Ram, Jerusalem 9190401, Israel*

§*Department of Chemical Research Support, Weizmann Institute of Science, Rehovot 7610001, Israel*

‖*Equal contribution*

E-mail: uri.raviv@mail.huji.ac.il

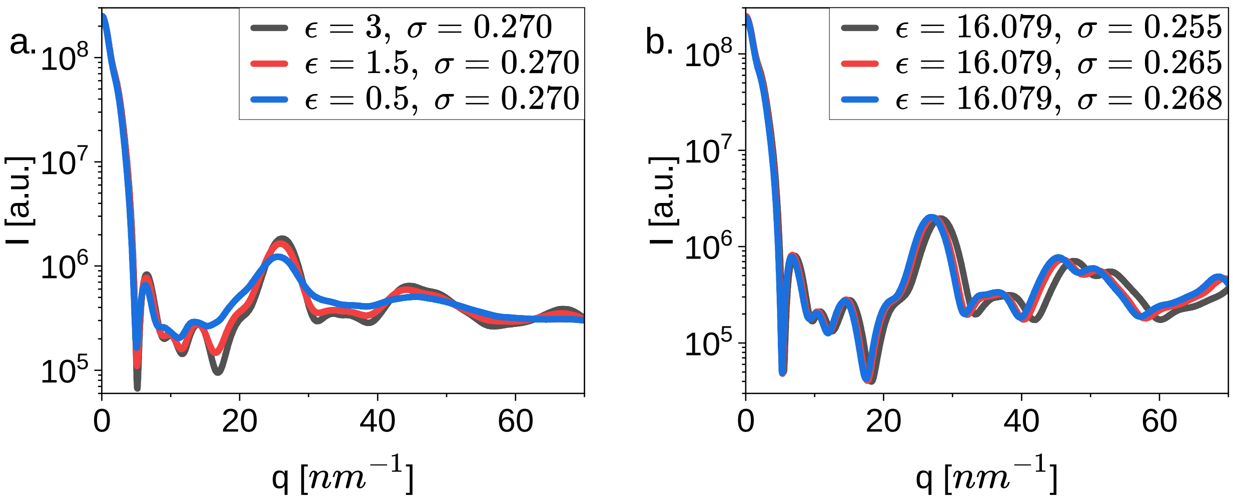

Figure S1: Effect of the Lennard-Jones potential parameter values used in the MC simulations on the 1D scattering curves of Au$_{144}$(p-MBA)$_{60}$[S1] a) Effect of the Lennard-Jones well depth, given by ϵ (in units of eV) at a fixed excluded volume term given by σ (in nm). b) Effect of varying the excluded volume term, σ (in nm), at a fixed Lennard-Jones well depth ϵ in $k_B T$ units (equal 0.4106 eV at room temperature).

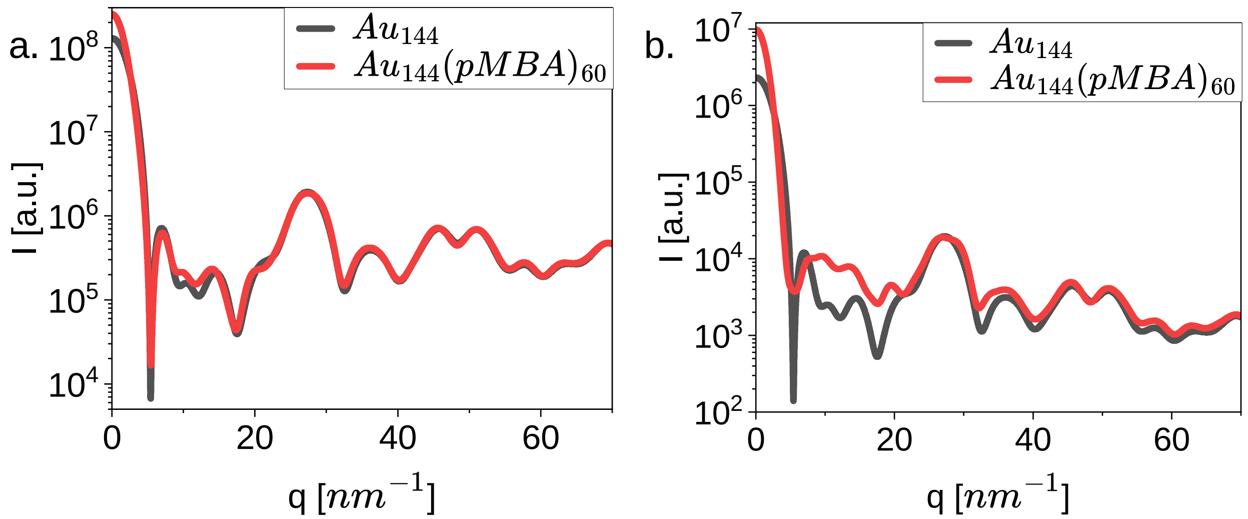

Figure S2: The expected difference in 1D scattering curves between Au$_{144}$(p-MBA)$_{60}$ with and without ligands in a) X-ray scattering and b) electron scattering. The electron density of the ligands is significantly lower than that of the gold core (Figure S1a), hence its contribution to X-ray scattering is negligible. The atomic potentials, however, are predicted to give larger differences in the electron scattering at an intermediate q-range.

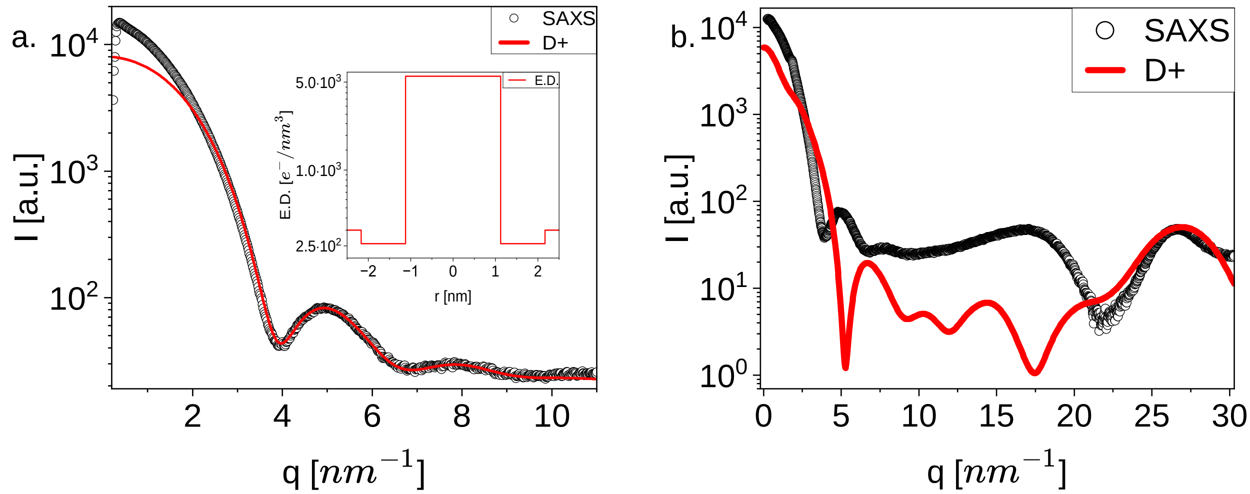

Figure S3: Background-subtracted azimuthally integrated solution wide-angle X-ray scattering from Au_{144}(p-MBA)$_{60}$ nanoparticles (black open circles in a and b) fitted to: a. Uniform spherical core-shell model (red curve), computed by X+ program.[S2] The core has a radius of 1.12 nm, a Gaussian polydispersity with $\sigma = 0.096$ nm and a mean electron density of 5560 e^-/nm^3. The shell has a thickness of 1.048 nm and a mean electron density of 260 e^-/nm^3 (and no polydispersity). The inset shows the radial electron density profile of the spherical core-shell model along the radial direction, r. b. The D+ computed model from Figure 6b (red curve).

References

(S1) Yan, N.; Xia, N.; Liao, L.; Zhu, M.; Jin, F.; Jin, R.; Wu, Z. Unraveling the long-pursued Au144 structure by x-ray crystallography. *Sci. Adv.* **2018**, *4*, eaat7259.

(S2) Ben-Nun, T.; Ginsburg, A.; Székely, P.; Raviv, U. *X+*: a comprehensive computationally accelerated structure analysis tool for solution x-ray scattering from supramolecular self-assemblies. *J Appl Crystallogr* **2010**, *43*, 1522–1531.
